# The Road to Re-Use of Spice By-Products: Exploring Their Bioactive Compounds and Significance in Active Packaging

**DOI:** 10.3390/foods14142445

**Published:** 2025-07-11

**Authors:** Di Zhang, Efakor Beloved Ahlivia, Benjamin Bonsu Bruce, Xiaobo Zou, Maurizio Battino, Dragiša Savić, Jaroslav Katona, Lingqin Shen

**Affiliations:** 1 School of Food and Biological Engineering, Jiangsu University, Zhenjiang 212013, China; d.zhang@ujs.edu.cn (D.Z.); efakorahlivia@gmail.com (E.B.A.); benjaminbonsubruce@ymail.com (B.B.B.); zou_xiaobo@ujs.edu.cn (X.Z.); 2 Joint Laboratory on Food Science, Nutrition, and Intelligent Processing of Foods, Jiangsu University, Polytechnic University of Marche, Universidad Europea Del Atlantico, 60130 Ancona, Italy; m.a.battino@staff.univpm.it; 3 Department of Clinical Sciences, Faculty of Medicine, Polytechnic University of Marche, 60130 Ancona, Italy; 4 Faculty of Technology in Leskovac, University of Niš, 16000 Leskovac, Serbia; savic@tf.ni.ac.rs; 5 Faculty of Technology Novi Sad, University of Novi Sad, 21000 Novi Sad, Serbia; jaroslav.katona@uns.ac.rs; 6 School of Chemistry and Chemical Engineering, Jiangsu University, Zhenjiang 212013, China

**Keywords:** spice by-products, bioactive compounds, biodegradable packaging, extraction, circular bioeconomy, active packaging

## Abstract

Spice by-products, often discarded as waste, represent an untapped resource for sustainable packaging solutions due to their unique, multifunctional, and bioactive profiles. Unlike typical plant residues, these materials retain diverse phytochemicals—including phenolics, polysaccharides, and other compounds, such as essential oils and vitamins—that exhibit controlled release antimicrobial and antioxidant effects with environmental responsiveness to pH, humidity, and temperature changes. Their distinctive advantage is in preserving volatile bioactives, demonstrating enzyme-inhibiting properties, and maintaining thermal stability during processing. This review encompasses a comprehensive characterization of phytochemicals, an assessment of the re-utilization pathway from waste to active materials, and an investigation of processing methods for transforming by-products into films, coatings, and nanoemulsions through green extraction and packaging film development technologies. It also involves the evaluation of their mechanical strength, barrier performance, controlled release mechanism behavior, and effectiveness of food preservation. Key findings demonstrate that ginger and onion residues significantly enhance antioxidant and antimicrobial properties due to high phenolic acid and sulfur-containing compound concentrations, while cinnamon and garlic waste effectively improve mechanical strength and barrier attributes owing to their dense fiber matrix and bioactive aldehyde content. However, re-using these residues faces challenges, including the long-term storage stability of certain bioactive compounds, mechanical durability during scale-up, natural variability that affects standardization, and cost competitiveness with conventional packaging. Innovative solutions, including encapsulation, nano-reinforcement strategies, intelligent polymeric systems, and agro-biorefinery approaches, show promise for overcoming these barriers. By utilizing these spice by-products, the packaging industry can advance toward a circular bio-economy, depending less on traditional plastics and promoting environmental sustainability in light of growing global population and urbanization trends.

## 1. Introduction

The growing global population and urbanization have heightened the significance of food packaging in preserving food quality and safety. The processed food industry now prioritizes innovative packaging materials that fulfill functional requirements while addressing environmental concerns associated with conventional plastic packaging, such as non-degradability and toxin leaching [1,2]. Active packaging, including edible films and coatings (EFCs), extends beyond merely containing food by incorporating bioactive compounds that interact with the food or surrounding environment. These packaging systems can enhance food stability, safety, and shelf life through moisture or oxygen absorption or controlled release of preservative compounds [3,4].

Historically, spices have played a dual role in food systems, both enhancing sensory appeal and naturally preserving foods through their inherent antimicrobial and antioxidant activities, thus prolonging shelf life. This traditional understanding provides the groundwork for their contemporary use in active packaging technologies [5]. The global spice market continues to expand, with the oleoresin segment alone reaching approximately USD 1.44 billion in 2018 [6,7,8,9], generating significant waste streams, including peels, seeds, leaves, and processing residues. When improperly managed, these by-products contribute to environmental challenges, including greenhouse gas emissions and soil degradation [10]. However, recent research reveals that these discarded materials possess valuable bioactive properties that could revolutionize sustainable packaging development through controlled release antimicrobial and antioxidant effects with environmental responsiveness to pH fluctuations, humidity variations, and temperature changes [11,12].

Unlike typical plant residues, spice by-products display exceptional retention of volatile bioactives following industrial processing. They also display enzyme-inhibiting properties and maintain thermal stability during processing. Recent studies have demonstrated their significant potential, with ginger and onion residues showing enhanced antioxidant and antimicrobial properties due to high phenolic acid and sulfur-containing compound concentrations [13,14], while cinnamon and garlic waste effectively improve mechanical strength and barrier attributes owing to their dense fiber matrix and presence of bioactive aldehydes [15,16].

Despite these advantages, several hurdles remain, including the long-term storage stability of certain bioactive compounds, achieving consistent mechanical properties during scale-up, managing compositional variations inherent in these by-products, and cost competitiveness with conventional packaging.

While existing literature has acknowledged the importance of spices and their role in food packaging systems, there is a notable gap in comprehensive reviews that focus on the transformation of spice by-products, including processing waste and residues, in the development of active food packaging. This review addresses the literature gap by examining how spice by-products are converted into packaging materials, focusing on their bioactive compound profiles and processing pathways. Hence, this review is structured into sections covering classification of waste and bioactive compounds, extraction methodologies, compound stability, and practical applications of spice by-products in active packaging development.

## 2. Classification, Extraction, and Stability of Bioactive Compounds in Spice By-Products

### 2.1. Spice By-Products and Their Bioactive Compounds

Spices comprise a diverse range of natural botanical products, including various parts of plants, such as fruits, seeds, flowers, roots, leaves, bark, and resins. This wide array reflects the rich complexity of spices and their essential role in culinary and medicinal applications [17,18]. Table 1 summarizes the by-products and waste parts obtained from some commonly known spices across the world.

Spice by-products are the residual materials generated during the processing of spices. These by-products include peels and skins, which are the outer layer of spices typically discarded during processing; seeds, which are the remnants of seeds from spices, such as coriander or cumin, after the extraction process; stems and leaves, which are plant portions that are not commonly used in the final spice product; and extraction waste/residue, which are leftover materials remaining after solvent, essential oil, and oleoresin extraction of spices. These materials are sometimes referred to as spent residue or spent spice. Another form of by-product known as herbal dust, according to scientists [31,32,33], is derived from the filter-tea industry and constitutes a specific fraction characterized by a mean particle size that is comparably smaller than the pores of the filter-tea bags (0.315 mm). Spice by-product portions are a rich composition of nutrition and several chemicals. Table 2 outlines the chemical composition of spice waste portions that are frequently used.

**Figure 1:**
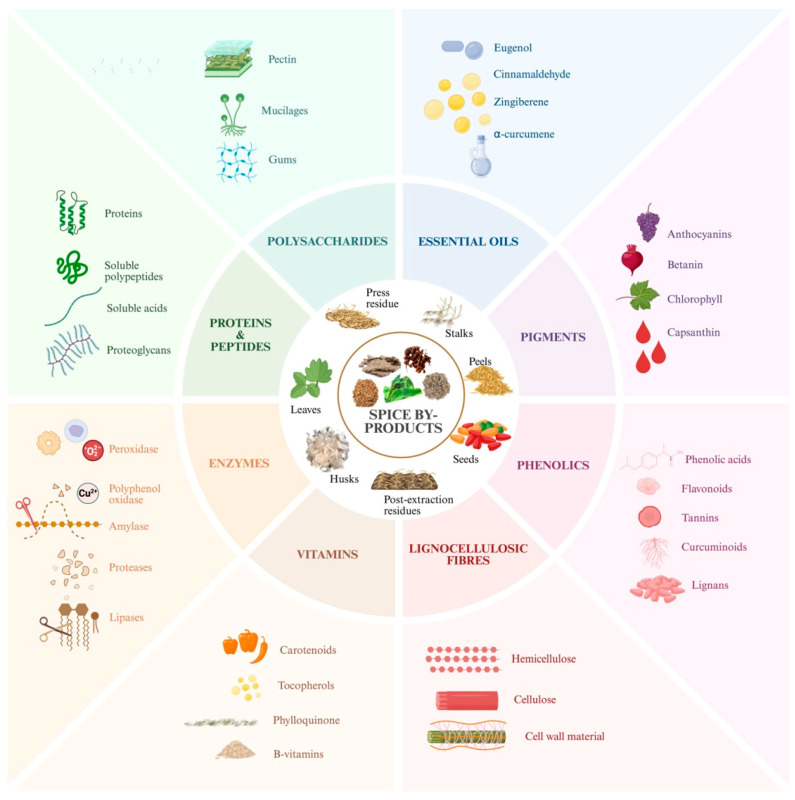
Classification of bioactive compounds derived from spice by-products. Created in BioRender. Zhang, D. (2025) https://BioRender.com/ucavrfv (accessed on 14 April 2025).

Bioactive compounds are substances that demonstrate biological activity and have positive effects on living organisms, such as lowering cholesterol levels or alleviating inflammation. These compounds are present in by-products and encompass a variety of types, including lipids, carbohydrates, phenolic compounds, proteins, and peptides [46]. Spice by-products constitute a great amount of bioactive compounds, which are being utilized in diverse ways in the food and beverage industry [47]. The bioactive compounds contained in spice by-products are distributed in the various parts of the plants, like the seeds, peels, flowers, straws, herbal dust, residual biomass, and sometimes the fruits, and have been summarized in Figure 1.

These bioactive compounds are not just an inherent component of the spice but largely influence the health-promoting properties of the whole product. In essence, knowing the bioactive composition can provide the necessary knowledge required for the further application of spice by-products in different spheres of industry. For example, foods containing allicin obtained from garlic skin may provide health benefits, such as managing blood sugar levels, anti-tumor, anti-fungal, and anti-microbial properties, providing a distinctive combination of flavor, nutritional value, and health advantages [48].

#### 2.1.1. Phenolic Compounds

Phenolic compounds (PCs), defined by their aromatic rings and hydroxyl groups, encompass more than 8000 identified structures, ranging from simple phenolic acids to intricate tannins. Researchers have reported that PCs are also among the most prominent bioactive components in spice by-products [49,50,51], ranging from polyphenols in pepper seeds [52] to flavonoids (flavonols, flavones, catechins, flavanones, anthocyanidins, and isoflavones), phenolic acids, stilbenes, coumarins, and tannins. Their structural diversity translates to their multifunctional potential in food packaging applications, where different phenolic classes contribute distinct protective mechanisms.

The distribution and concentration of PCs in spice by-products follow predictable patterns based on plant morphology and metabolic function. Phenolic acids, a principal category of these chemicals, are frequently found in protective plant tissues—seeds, skins, and leaves—where they exist primarily as bound forms (amides, esters, and glycosides [53].

While requiring specific extraction approaches, this bound nature enhances stability and enables controlled release in packaging systems. Coriander seeds exemplify this potential, containing 0.5119–2.6297 g GAE/100 g total phenolics and 0.2315 to 0.6280 g catechin equivalent/100 g flavonoid [54]. Recent research has investigated the extraction of oils from spice processing by-products due to their high phenolic content to incorporate them into diverse products and formulations as natural antioxidants. Notable among them is the recent investigation conducted by Nambiraj et al. [55], who found that essential oil extracted from *Curcuma longa* L. (turmeric) leaves demonstrated the best antioxidant capacity compared to three other oils due to its PC constituents, turmerone (20.5%) and 1,8-cineole (8.89%). Similar volatiles were also isolated from the essential oils of the press residue obtained after turmeric juice extraction, major constituents comprising ar-tumerone, curlone, β-sesquiphellandrene, and zingiberene [19]. The top and bottom bulbs of onions have been reported to contain quercetin, a potent polyphenol, whereas the major PCs isolated from garlic skins were caffeic, ferulic, di-ferulic acids, and hydroxybenzoic acid. Garlic peels are a rich source of anthocyanins, these act as antioxidants and inhibit food oxidation [56]. Anthocyanins, such as cyanidin 3-O-glucoside, a constituent of the polyphenolic compound family present in *Zanthoxylum bungeanum*, contributes to the crimson pigmentation observed in the fruit peel of the plant [57]. Processing conditions critically affect phenolic stability and bioactivity. For instance, thermal treatments of ginger converting gingerols to shogaols, demonstrates how controlled transformations can enhance specific activities, while potentially compromising stability. Additionally, the formation of zingerone during storage introduces variability in bioactivity [58]. This sensitivity has driven the development of advanced extraction techniques, including subcritical water extraction (SWE) and ultrasound-assisted extraction (UAE) with deep eutectic solvents, which preserve heat-sensitive compounds while maintaining structural integrity. For glycosylated phenolics, like miquelianin and robinobiosides, UAE with deep eutectic solvents (DESs) enables efficient extraction and controlled release—an asset for intelligent packaging systems [59,60]. Understanding these chemical relationships guides the strategic selection of spice by-products for packaging applications. Table 3 presents the variety of PCs identified in frequently utilized spice by-products. Rutin, which can be sourced from multiple spices, provides both protection against oxidation and UV rays. On the other hand, eugenol, found in the residues of clove and cinnamon, possesses powerful antimicrobial characteristics that make it ideal for packaging perishable items. Garlic, onion, turmeric, and clove by-products demonstrate the most promising phenolic profiles for active packaging applications, balancing extractability, bioactivity, and functionality.

#### 2.1.2. Polysaccharides

Polysaccharides are macromolecules composed of monosaccharides linked via glycosidic bonds, with varying properties according to content, linkage type, configuration, polymerization degree, and molecular weight. Cellulose, pectin, and starch exemplify these compounds, which demonstrate therapeutic effects, including immunomodulatory, antiviral, antibacterial, antioxidant, and anticancer activities [75]. Hao et al. [76] conducted a thorough analysis demonstrating that the polysaccharides present in ginger peel are its most abundant bioactive components. Plant polysaccharides have a significant propensity to interact with various bioactive components, enhancing functional characteristics. These interactions can improve the polysaccharides’ bioavailability, efficacy, and overall benefits, making them more effective in various applications, including food packaging systems. A clear validation was recorded by Qui et al. [77], who optimized the modification of ginger peel polysaccharides (GPPs) with chromium (III) [Cr (III)] to enhance their anti-inflammatory properties. The chelation process improved the molecular weight, thermal stability, and glucose content while preserving their structure. In a zebrafish inflammation model, GPP-Cr (III) complexes showed better anti-inflammatory effects than unmodified GPPs by regulating the MyD88/NF-κB/MAPK/iNOS pathways. These findings suggest that GPP-Cr (III) complexes could serve as functional food components and dietary Cr (III) supplements, promoting the use of ginger peels. Also, recent investigations have demonstrated that by-products derived from onions and garlic contain various polysaccharide fractions, including galactose, rhamnose, galacturonic acid, arabinose, fructose, and glucose [78,79]. Onion skins have been reported to contain a decent concentration of fructo-oligosaccharides and pectin, a major cell wall component, amidst other components, such as hemicellulose and cellulose [80]. Kallel et al. [81] isolated a novel polysaccharide from garlic straws using hot water extraction, comprising glucose, galactose, xylose, and mannose. Furthermore, the seeds and leaves of Szechuan pepper are composed of polysaccharides, including rhamnose, arabinose, glucose, xylose, and galactose [69]. These composition data are essential for understanding their potential applications in biodegradable food packaging systems.

#### 2.1.3. Proteins, Peptides, and Enzymes

Spice by-products contain significant protein fractions that yield bioactive peptides (BPs) upon enzymatic hydrolysis. The functional efficacy of these peptides correlates inversely with chain length; shorter peptides (<800 Da) demonstrate superior bioactivity compared to longer chains [82,83]. While leguminous plants traditionally dominate BP research, researchers have found that proteins and peptides constitute a great proportion in seeds, leaves, meals and caky residues or powders, and stems of commonly used culinary spices [84,85,86], making them a highly valuable asset within the food and pharmaceutical industries. Analysis of protein distribution across spice by-products reveals strategic valorization opportunities; garlic tissues (10% protein) yield sulfur-containing peptides, including S-allyl cysteine, that enhance antimicrobial properties [87,88]. Pepper seeds also comprise 13.8–28.3% protein with albumins and glutelins comprising over 75% of this fraction [89]. In recent studies, researchers have shown that waste spice, such as chopped pepper seeds contain 17.3% crude protein along with 17 identified amino acids [90]. Furthermore, red pepper seed meal, which is a by-product of red pepper oil extraction, may have an even higher protein content, reaching as much as 26% crude protein with elevated lysine, threonine, and tryptophan levels [91]. Similarly, spent ginger along with its stems and leaves contain 7–9% protein [34]. Notably, onions store approximately 7% proteogenic amino acids that redistribute during ripening, with glutamine and arginine predominating [92].

Beyond nutritional applications, oxidative enzymes extracted from spice processing waste demonstrate significant potential for biodegradable packaging systems. Stems, leaves and oilseeds are a suitable habitation for plant lipases [93]. Peroxidases (POD) isolated from onion bulb trims [94] and polyphenol oxidases (PPO) purified from turmeric rhizomes could facilitate controlled biodegradation of packaging materials while potentially extending shelf-life through targeted oxidation management [95].

Simultaneously, spice by-products exhibit enzyme-inhibiting properties that prevent food deterioration. Extracts from star anise and coriander waste demonstrate anti-tyrosinase activity, preventing enzymatic browning, while capsaicin and dihydrocapsaicin from chili waste inhibit α-glucosidase and α-amylase through competitive mechanisms [96,97,98].

This dual enzymatic capability creates intelligent packaging systems where beneficial enzymes promote controlled material degradation while inhibitory compounds selectively prevent unwanted food enzymatic reactions, enabling comprehensive management of both packaging biodegradation and food preservation.

#### 2.1.4. Essential Oils

Essential oils in spice by-products represent complex bioactive matrices comprising terpenes, alcohols, esters, aldehydes and phenols that demonstrate multifunctional biological activities against microbes and oxidative processes [99,100,101]. Their structural diversity and functional versatility make them particularly valuable for use in the food packaging sector.

Extraction methodology significantly influences the essential oil yield and bioactivity profile. Conventional approaches, like steam distillation, solvent extraction, and hydrodistillation, present specific limitations, such as thermal degradation of volatile compounds, potential solvent contamination, and impaired functional properties [102,103]. This relationship between extraction technology and bioactive quality necessitates optimization protocols that preserve antimicrobial efficacy for packaging applications.

Coriander’s aerial parts, especially its leaves and stems, are rich in essential oils, comprising approximately 24 compounds (98% of the oil content). Ghasemi et al. [104] discovered that among the primary constituents are *cis*-phytol, which imparts a fresh, grassy aroma; decanal and dodecanal, known for their citrusy fragrances; and *n*-decanol, 1-tetradecanol, *trans*-2-undecen-1-ol, methyl chavicol, which add to coriander’s rich and appealing aroma [105].

Chemical composition analysis reveals distinct essential oil profiles across different spice by-products. For instance, Rawat et al. [106] identified that the Zingiberaceae family, particularly *Zingiber zerumbet* (red ginger), illustrates significant variability in essential oil composition obtained from hydrodistillation across its roots, rhizomes, cones, and leaves. While all parts contain oxygenated sesquiterpenes, the specific compounds differ greatly. Zerumbone, a significant terpene, is abundant in the roots and rhizomes but absent from the leaves and cones. Conversely, (*E*)-nerolidol predominates in the leaves and cones, while linalool and β-caryophyllene are significant in the cones and leaves, respectively, but minimal or absent elsewhere. This variability in essential oil composition is not unique to ginger; it is a characteristic feature shared by many spice species [55,107,108]. Turmeric leaves provide monoterpene-rich oils, especially 40.19% myrcene and 23.05% *p*-cymene. Also, turmeric industrial residue obtained from the turmeric processing industry yields volatiles, such as ar-turmerone, turmerone, and curlone [109]. Even though essential oils are present in most spice by-products, Kita et al. [110] in Poland have reported that no direct correlation exists between essential oil composition and volatile organic compound (VOC) emission profiles, highlighting the complexity of incorporating these compounds into functional packaging systems.

However, Ma et al. [111] highlighted that this lack of direct correlation arises from complex interactions within EO mixtures, where VOCs exhibit synergistic or antagonistic effects that alter emission dynamics. VOC release is further complicated by environmental factors critical to food packaging—including temperature fluctuations, humidity variations, and pH changes—which significantly affect diffusion rates and bioactivity predictability.

Consequently, effective packaging design requires functional emission profiling that links real-time headspace VOC concentrations with antimicrobial performance, rather than relying solely on static EO composition [112]. Future developments should focus on matrix engineering and controlled release technologies that optimize dynamic emission characteristics for specific storage conditions, enabling predictable bioactivity through integrative chemical–environmental–functional design approaches.

#### 2.1.5. Pigments

Spice by-products yield diverse natural pigments that enhance the visual appeal and functional properties of biodegradable packaging systems. These compounds extend beyond aesthetic contributions to provide significant bioactive benefits, including antioxidant, antimicrobial, and anti-inflammatory properties [113]. Pigment distribution analysis across spice processing residues reveals strategic extraction opportunities. Onion peels yield carotenoids, including β-carotene, phytoene, torulene, and torularhodin, through sustainable microbial synthesis [114], while onion anthocyanins are widely distributed in its skin [14,92,115,116]. Saffron floral residues provide crocin, crocetin, safranal, and picrocrocin [117]. The composition of pepper seed oils was analyzed by Chouaibi et al. [118] and contained zeaxanthin, lutein, β-cryptoxanthin, capsorubin, capsanthin, and other carotenoids with extraction yields highly dependent on processing methodology.

Cinnamon bark waste contains tannins and cinnamaldehyde, expanding the palette of natural colorants available from spice processing streams [119]. These pigments offer dual functionality in packaging applications, i.e., visual indication of freshness/quality while providing bioactive protection. Integrating these compounds into packaging materials enables the development of systems that communicate product status while extending shelf life.

#### 2.1.6. Dietary Fiber

In addition to the visual attributes and bioactive benefits imparted by pigments from spice by-products, the fibrous components of spice by-products represent another essential class of functional components. Spice by-products contain structurally diverse fiber fractions, including both soluble (pectin, beta-glucans, gums, and mucilage) and insoluble (cellulose, hemicellulose, and lignin) [120]. The chemical composition of dietary fiber exhibits a substantial correlation with its functional properties, thereby contributing significantly to the nutritional and functional properties of spice by-products.

Cvetković et al. [121] discovered that the primary component of the carbohydrate content in pepper seeds is dietary fiber. Red pepper seeds can be considered a sustainable source of dietary fiber, with significant levels ranging from 26% to 61%. According to Sowbhagya, H.B. [15], spice spents, the by-products from spice oil or flavor extraction, are high in dietary fiber, comprising up to 62% of the total nutritional composition. This surpasses the fiber content found in many fruits and vegetables, showcasing their potential as a valuable dietary component. The researcher identified spent spices, such as ginger, garlic, chili pepper, cumin, turmeric, coriander, and clove, as significant sources of dietary fiber. Also, according to the analysis of proximate composition by Mondal et al. [122], a quantity of 100 g of curry leaves contains 6.8 g of crude fiber. The yields of insoluble and soluble dietary fiber ranged from approximately 60% to 69% and 10% to 20%, respectively. The higher the soluble dietary fiber concentration, the more enhanced the water-holding and gelling capacities, improving the texture and mouthfeel of various products. On the other hand, as the insoluble dietary fiber increases, the more stable the fiber, giving a more desirable crunch and adding value to targeted formulations. Dietary fibers have been proven to further enhance product stability and texture in the food industry.

### 2.2. Extraction of Spice By-Products’ Bioactive Compounds for Packaging Applications

Extraction techniques play a crucial role in recovering bioactive compounds from spice by-products. Spice by-products contain valuable components that enhance packaging functionality while supporting circular economy principles [123,124]. Traditional extraction methods, like hydrodistillation and maceration, typically require significant energy and toxic solvents, contradicting the sustainability goals of modern food packaging systems.

Anastas and Warner, however, emphasized the efficient utilization of raw materials, the avoidance of toxic reagents, and the promotion of health and environmental safety. To adhere to these principles, the extraction of bioactive materials should employ sustainable and cost-effective methodologies. Deep eutectic solvent (DESs) exhibit low toxicity, environmental friendliness, biodegradability, and recyclability [125,126], therefore is a sustainable alternative to harmful conventional solvents [127,128]. DES consists of hydrogen-bond acceptors (HBA), such as quaternary ammonium salts, and hydrogen-bond donors (HBD), like alcohols and carboxylic acid [129]. Natural deep eutectic solvents (NaDESs) derived from natural sources enhance packaging sustainability [130].

Safety and regulatory considerations for DESs in food packaging applications require comprehensive evaluation. DESs have lower acute toxicity than conventional organic solvents, with LD50 values over 2000 mg/kg for choline-based systems yet their use in food contact materials requires thorough migration studies and toxicological assessments. Current regulatory frameworks, including EFSA guidelines and FDA food contact substance regulations, require extensive safety data for novel solvents, including genotoxicity studies, 90-day sub-chronic toxicity tests, and specific migration limits not exceeding 10 mg/kg food simulant for most DES components. Residual DES in final packaging for food contact must be below 50 ppm, necessitating thorough purification within regulatory frameworks [131,132].

The regulatory landscape for bioactive packaging materials has evolved significantly since the foundational EC 1935/2004 and EC 450/2009 regulations. Current safety assessments follow established frameworks from FAO and EFSA, employing quantitative risk assessment methodologies and specific migration testing standards, such as ISO 15161 [133] and CEN/TS 14234 [134,135]. Recent biopolymer restrictions by the European Chemicals Agency further emphasize the regulatory push toward natural alternatives, like spice by-product extracts, although these materials require equally rigorous safety evaluation and migration assessment protocols [136].

Several studies have reported the maximum and best bioactive compounds’ recovery amounts using DESs, including rich extracts from ginger powder and herbal dust [137,138], curcumin from turmeric, cinnamon, sesame, and curry powder [139], and lignin fractions from garlic skins [140]. Advanced green extraction methods for packaging applications include supercritical fluid extraction (SFE), ultrasound-assisted extraction (UAE), microwave-assisted extraction (MAE), enzyme-assisted extraction (EAE), subcritical water extraction (SWE), pulsed electric field extraction (PEF), high pressure processing (HPP), pressurized liquid extraction (PLE), cold plasma technology and hydrodynamic cavitation (HC). Each method necessitates the validation of process parameters to ensure that extracted compounds meet food safety standards and do not introduce harmful by-products or degradation compounds.

HC, for instance, employs pressure variations to create cavitation bubbles that generate energy capable of disrupting plant cell walls, efficiently releasing bioactive compounds while preserving their functional properties. Combined techniques often show better results for packaging applications. In a study conducted by Zhu et al. [141], a cocktail of enzyme-assisted extraction and NaDES choline was employed to enhance the recovery of capsaicin from chili pepper seeds, achieving yields up to 10.65 ± 0.14 mg/g at scale-up; compounds subsequently incorporated into antimicrobial packaging films with enhanced preservation properties. In 2024, Sulejmanović et al. [142] used supercritical carbon dioxide to extract ginger herbal dust bioactives, and its pharmacological activity in vitro and in silico was analyzed. In addition, Sagar et al. [80] revealed that employing ultrasound-assisted extraction and lactic acid and glucose as green solvents was effective in isolating tyrosol, rutin hydrate, and caffeic acid as significant compounds in onion seed production by-products constituting umbels and dry scapes, hence contributing to the valorization of this residue. The toxicological profile of phenolic compounds, such as tyrosol and caffeic acid, requires evaluation for packaging applications. Although known for their antioxidant properties, their safety depends on migration levels and exposure assessments. EFSA classifies caffeic acid as acceptable for food use with a migration limit of 30 mg/kg, while tyrosol requires an individual assessment based on its intended use [143]. The extraction method directly impacts both the functionality and biodegradability of the resulting packaging material, as extraction residues and solvents can affect material properties. Furthermore, the choice of extraction method influences the safety profile of the final packaging material, as residual solvents, extraction by-products, and compound degradation can introduce potential safety concerns that require comprehensive risk assessment according to current food contact material regulations [144]. Selecting the appropriate green extraction methodology is necessary for developing packaging that aligns with circular economy principles and addresses growing regulatory requirements for food-contact materials.

### 2.3. Stability of Spice By-Products’ Bioactive Compounds for Enhanced Packaging Functionality

**Figure 2:**
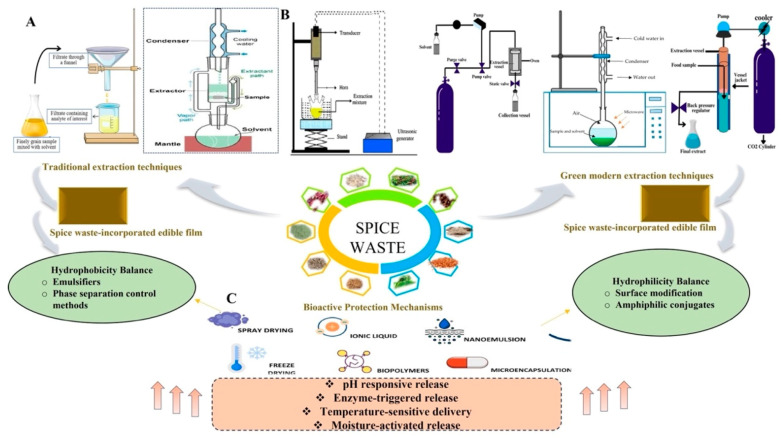
Extraction and improved stability of bioactive compounds from spice by-products. (**A**) Traditional extraction methods, including maceration and Soxhlet extraction; (**B**) Green extraction techniques: ultrasound-assisted extraction, pressurized liquid extraction, supercritical fluid extraction, and microwave-assisted extraction; (**C**) Bioactive compound stabilization mechanisms in food packaging systems. Reproduced with permission [146,147].

Spice by-products represent a highly diverse group characterized by considerable variation in their chemical composition, physical properties, and stability profiles. Bioactive compounds obtained from these by-products exhibit unique chemical structures with varying responses to environmental conditions, like light, temperature, and pH. For active packaging applications, maintaining the stability of these compounds throughout the packaging lifecycle is essential for sustained antimicrobial, antioxidant, and shelf-life extension properties [145]. These compounds typically face challenges, including poor solubility, low bioavailability, and susceptibility to degradation that must be addressed to ensure packaging effectiveness. These challenges can be addressed by combining extraction techniques and innovative protection mechanisms as illustrated in Figure 2.

Encapsulation technologies enable the controlled release and protection of bioactive compounds from spice by-products in packaging applications through physical methods (spray drying, freeze drying, fluidized bed coating, electrospinning), chemical approaches (ionotropic gelation), and physicochemical approaches (coacervation) [148]. These systems preserve functional properties during manufacturing and storage while ensuring compatibility with biodegradable packaging matrices [149].

Hydrophobic compounds, such as curcumin, capsaicinoids, gingerol, and allicin, require specialized delivery systems, with liposomes proving particularly effective for encapsulating water-soluble, lipid-soluble, and amphiphilic molecules [150]. Current research focuses on stimuli-responsive nanocapsules that react to pH, temperature, or microbial presence for active packaging, utilizing advanced techniques, like microfluids, for enhanced size control and release characteristics [151].

## 3. Spice By-Products in Food Packaging Systems

### 3.1. Biodegradable Films: Regulatory Framework and Environmental Impact

Biodegradation, the chemical breakdown of materials through metabolic or enzymatic activity of microorganisms under aerobic and anaerobic conditions, represents a critical solution to packaging waste persistence [152]. This natural decomposition process depends upon molecular structure and chemical composition [153,154,155].

Biobased polymers derived from biological materials, including spice processing residues, offer superior biodegradability compared to petroleum-based synthetic polymers, which create significant environmental issues through recycling complexities and contribute to solid waste streams. Current regulatory frameworks in major markets are increasingly mandating that packaging materials demonstrate actual biodegradability under specified environmental conditions. Emerging regulations are addressing concerns about “greenwashing” through standardized testing methods and certification requirements. Regulatory trends significantly affect the choice and development of packaging materials, especially for active packaging systems.

Packaging materials incorporating spice by-products must be designed to balance functionality with biodegradation timelines appropriate for their intended applications and disposal environments. Biodegradable films utilizing spice by-product components significantly reduce environmental persistence while potentially enhancing food preservation through bioactive properties. Polysaccharides, particularly lignocellulose fibers from spice by-products, create films with enhanced protective features while maintaining rapid biodegradation [156,157]. These new materials turn food packaging into sustainable options that help with environmental issues and meet regulatory concerns.

#### 3.1.1. Spice By-Products as Sources of Lignocellulosic Fibers for Biodegradable Packaging

Spice processing by-products represent valuable sources of lignocellulosic fibers for biodegradable food packaging applications. These materials, including spent biomass, husks, stems, and other processing residues, provide sustainable alternatives to synthetic reinforcing agents while addressing waste management challenges. The fibers derived from these materials, rich in cellulose, hemicellulose, and lignin, significantly improve mechanical properties of biodegradable packaging films based on starch, polylactic acid (PLA), and protein matrices [158].

Incorporating natural fibers from spice by-products into packaging systems offers dual benefits: diverting agricultural waste from disposal while enhancing material performance to meet technical requirements for food packaging applications [159].

Key mechanical parameters for packaging materials include tensile strength, stress, strain, and Young’s modulus, all of which can be optimized through proper selection and processing of spice-derived fibers [160,161]. Cellulose from spice by-products comprises both flexible and rigid polymer components that create balanced mechanical profiles suitable for various packaging applications when integrated into biodegradable matrices. Numerous studies have demonstrated that cellulose nanofibers from these materials exhibit particularly advantageous mechanical properties while maintaining the transparency and biodegradability required for modern sustainable packaging [162].

#### 3.1.2. Processing Methods for Spice By-Product Fibers

Obtaining natural fibers from spice by-products requires methods that maintain their characteristics and guarantee biodegradability. Mechanical extraction is one efficient technique that physically divides fibers through regulated grinding. Chemical approaches using NaOH effectively remove non-cellulosic components, leaving cellulose-rich fibers with enhanced interfacial properties for biodegradable matrices. Acid and alkali hydrolysis represent widely employed techniques for fiber isolation, but these methods must be carefully controlled to preserve biodegradability [163]. Enzymatic treatments are less common but provide clear benefits for creating highly purified fibers by breaking down unwanted parts. Recent advancements show that these methods can be applied in practical ways. In 2023, researchers successfully isolated cellulose from ginger pulp through optimized chemical extraction and fabricated transparent, flexible, all-cellulose biodegradable films that exhibited exceptional mechanical and thermal characteristics while maintaining complete biodegradability [164].

Cellulose and derivatives extracted from spice by-products, including nanocrystals, microcrystalline cellulose, and modified celluloses, have demonstrated significant potential in biodegradable food packaging applications [163,165,166]. Successful implementations include cellulose extracted from garlic husks and skins [48,167,168], garlic stem [169], garlic straw [25], ginger and its stem [170,171], ginger agro-residues [172], and pepper seed pomace [40] for various biocomposite packaging applications.

#### 3.1.3. Mechanical Properties and Processing Techniques for Biodegradable Packaging

Studies suggest that fibers obtained from by-products of spices present unique mechanical benefits for packaging uses. Fibers from cinnamon bark exhibit significant tensile strength and rigidity because of their elevated cellulose levels [173], whilst ginger pomace fibers provide flexibility and durability [174]. After processing, ginger pulp maintains valuable fibrous characteristics, featuring starch-rich tissue and intact cellulosic structures that contribute to enhanced mechanical properties in resulting packaging materials. The analysis of ginger waste bio-films demonstrates that viscosity increases from 217 to 376 mPa·s with additional grinding cycles and decreased particle size. These films exhibit impressive mechanical properties with a maximum tensile strength of 15 MPa and strain of 4.8%. Food packaging applications remain thermally stable up to 150 °C, with degradation beginning only at temperatures exceeding 300 °C—characteristics that ensure packaging integrity under regular storage and distribution conditions [174].

**Figure 3:**
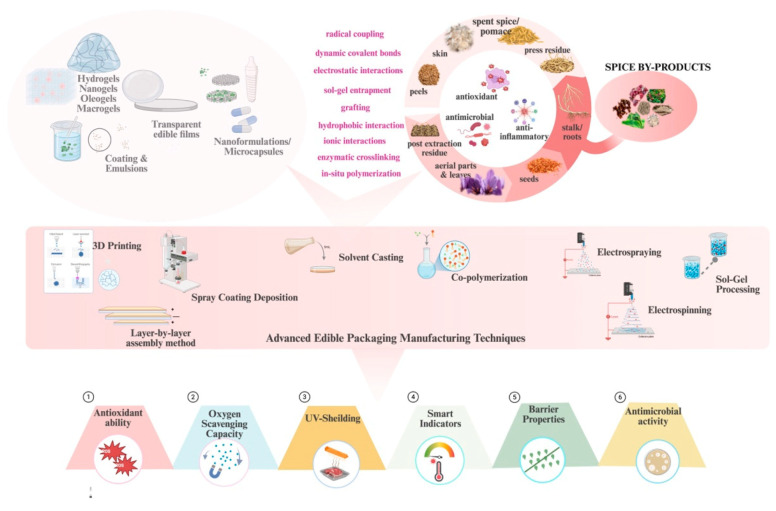
Advanced preparation methods of spice waste incorporated food packaging films for enhanced functionality. Created in BioRender. Zhang, D. (2025) https://BioRender.com/n74m448 (accessed on 22 May 2025).

Spice-derived fibers are incorporated into polymer matrices through various manufacturing processes, including solution casting, extrusion, and compression molding. In solution casting, fibers are suspended in a polymer solution before controlled drying [175]. Extrusion combines fibers with polymers at elevated temperature and pressure to produce reinforced films with enhanced properties. Additional fabrication methods for functional packaging include electrospinning, 3D printing, supercritical fluid technology, and spray drying (Figure 3), each offering specific advantages for particular packaging applications [176].

Cinnamon fibers integrated into starch-based films have increased tensile strength, reduced brittleness, and improved moisture resistance, showcasing the potential of spice by-products in enhancing biodegradable packaging solutions [16].

### 3.2. Edible Coatings

Edible coatings represent sustainable alternatives to conventional packaging by forming consumable protective barriers directly on food surfaces. These bio-based films extend the shelf life by controlling moisture transfer, oxygen exposure, and microbial growth—benefits particularly valuable for highly perishable produce [177]. Effective coating formulations balance barrier performance with sensory acceptability while maintaining regulatory compliance for food-contact materials. Edible coatings support circular economy principles by incorporating by-products from spice processing, simultaneously reducing food waste and plastic dependence. These coatings are formulated with hydrocolloids, lipids, and composite blends optimized for specific food preservation needs, providing eco-friendly alternatives to conventional packaging while meeting growing regulatory requirements for sustainable material [178,179].

Composite materials in edible coatings utilize various biopolymers derived from spice by-products, with hybrid formulations demonstrating superior barrier properties, mechanical strength, and environmental benefits compared to single-component coatings. Adding natural antimicrobial compounds from spice processing waste enhances the protective capabilities of these materials, effectively reducing spoilage and extending the shelf life of perishable foods [180]. Essential oils extracted from spice by-products represent key functional components for edible coatings. According to Ungureanu et al. [181], essential oil-based coatings can modify food flavor profiles through controlled migration of volatile compounds. Scientists [182,183] have confirmed these effects using cinnamaldehyde from cinnamon leaves encapsulated in chitosan coatings applied to meat and fruit products. These applications created modified atmospheres around the foods that effectively controlled microbial growth while masking undesirable off-flavors, demonstrating the multifunctional benefits of spice by-products in edible coating applications.

### 3.3. Pickering and Nanoemulsions

Pickering and nanoemulsions represent advanced colloidal systems with significant potential for active food packaging applications. These systems utilize solid particles or nanoscale droplets to stabilize emulsions, providing enhanced stability, controlled release of bioactive compounds, and improved barrier properties essential for practical food preservation [184]. Bioactive compounds from spice by-products, including polyphenols, essential oils, and flavonoids, serve as sustainable and functional ingredients for developing these advanced delivery systems [185,186,187].

Recent studies have explored applications of such by-products in stabilizing Pickering emulsions, where solid particles provide stability against coalescence, rather than traditional surfactants. Due to their surface-active properties, bioactive compounds from these materials effectively function as natural emulsifiers. Cellulose nanocrystals (CNCs) extracted from spice processing waste stabilize emulsions by forming protective layers around oil droplets, minimizing interfacial tension and preventing coalescence [188,189].

Spice-derived materials can create stable emulsions for packaging, enhancing structural integrity and mechanical properties, and improving barrier functions and protection. For example, incorporating cinnamon leaf essential oil into chitosan/zein-based films significantly enhances their antioxidant and antimicrobial properties [190], making them particularly useful for preserving sensitive food items, such as mushrooms.

## 4. Value-Added Benefits of Spice By-Products in Active Packaging

### 4.1. Antioxidant Packaging Systems

#### 4.1.1. Mechanism of Action

Antioxidant compounds derived from spice by-products represent a sustainable approach to enhancing food packaging functionality by mitigating oxidative degradation processes. In active packaging systems, these compounds interrupt lipid peroxidation and free radical propagation that would otherwise compromise food quality and shelf-life [191]. Aqueous and alcoholic extracts, essential oils, and polyphenolic concentrates obtained from spice processing residues provide effective antioxidant activity through hydrogen atom donation to free radicals, neutralizing them before they can initiate oxidative cascades within packaged foods. Active packaging incorporating these compounds functions through two primary mechanisms; release-type systems gradually transfer antioxidant compounds to food surfaces [192]. In contrast, scavenger-type systems intercept oxidative species within the packaging headspace. This dual approach allows packaging designers to address both immediate oxidation concerns and provide extended protection throughout the product shelf life while utilizing waste materials from spice processing.

#### 4.1.2. Sources and Efficacy

The increasing consumer demand for natural food additives has driven research toward utilizing spice by-products as sources of natural antioxidants [193]. These antioxidants, when incorporated into packaging materials, actively inhibit oxidation processes that lead to food deterioration, extending shelf life while maintaining product quality.

Antioxidant efficacy in packaging applications is quantified through standardized methodologies, including DPPH and ABTS radical scavenging assays, peroxide value measurements, and spectrophotometric phenolic content evaluations [179]. These analytical techniques enable researchers to identify optimal spice by-product extracts and develop effective antioxidant packaging systems with consistent performance. The functionality of these packaging systems depends primarily on the concentration and type of polyphenolic compounds present in spice by-product extracts. These compounds function as reducing agents within the packaging matrix, scavenging free radicals that would otherwise trigger oxidative deterioration in packaged foods [194]. This relationship between hydroxyl and carbonyl-rich compounds in spice residues and their stabilizing properties makes them particularly valuable for developing sustainable antioxidant packaging.

#### 4.1.3. Major Spice By-Products in Antioxidant Packaging

Several spice by-products demonstrate exceptional potential for antioxidant packaging applications. Ginger by-products offer remarkable antioxidant properties for active packaging applications, with decoctions and infusion extracts showing oxidative inhibition of up to 55% [195].

Ginger peel extracts, rich in rutin, zingerone and quercetin, provide excellent oxidation–reduction capabilities when incorporated into packaging films [196]. Additionally, ginger leaves and starch have shown promising antioxidant activities [13,197].

Onion by-products have been effectively transformed into functional packaging components that extend the shelf life of perishable foods. Researchers have incorporated onion peel and stalk extracts into sodium-alginate-carboxymethyl cellulose films, increasing the radical scavenging ability from 39% to 88% [198]. These enhanced protective capabilities enable the packaging to maintain food quality through natural preservation mechanisms. Red onion skin extracts exhibit exceptionally high antioxidant activity in packaging applications, with FRAP and ORAC values reaching 2017.34 μmol-equiv. Trolox per gram of dry weight [199] A study by Pirsa et al. [200] confirmed this by modifying a carboxymethylcellulose film with red onion peel extract and powder and boron nitride nanoparticles, and an increase in the antioxidant property of the film was observed.

Thyme and other spice by-products provide complementary antioxidant functions in packaging systems. Recent research has revealed that herbal dust from thyme filter-tea production contains 242 polyphenolic compounds, with carvacrol as the predominant monoterpene contributing to antioxidant performance in packaging applications [74,201].

Additional spice by-products demonstrating significant antioxidant potential in packaging include black pepper pericarp [202], *Capsicum* flavonoids [203], Szechuan pepper leaves [204], waste clove leaves [205], cinnamon bark extract [206], cinnamon leaf oil [207], paprika oleoresin [208], coriander seeds and residues [209], and rosemary [210].

This collectively demonstrates that residues from spices are not a waste, as when valorized efficiently, they hold great promise for developing sustainable and active packaging solutions while maintaining a circular economy and advancing toward a zero-waste global environment.

### 4.2. Antimicrobial Packaging Systems

**Figure 4:**
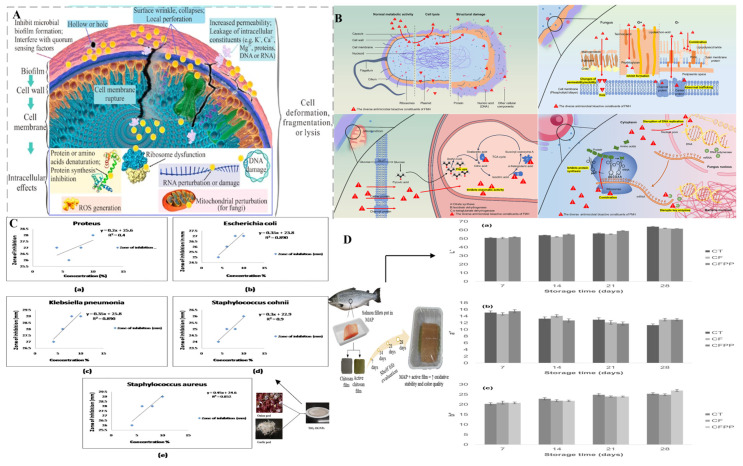
Antimicrobial mechanism and applications of spice by-product antimicrobial agents against food-borne pathogens. (**A**) Primary release antimicrobial mechanism of spice by-product bioactive compounds. Reproduced with permission [212] (**B**) Disruption and inhibition process of spice by-products bioactive compounds against cell membrane and DNA. Reproduced with permission [213] (**C**) Antimicrobial capacity of garlic and onion peel nanoparticles against bacterial pathogens including *Proteus*, *E. coli*, *Klebsiella pneumonia*, *Staphylococcus cohnii*, and *Staphylococcus aureus*. Reproduced with permission [216] (**D**) Color stability maintenance of salmon fillets by pepper residue-chitosan films over a period of 28 days. Reproduced with permission [217].

Antimicrobial packaging is required to safeguard food against microbial spoilage during preservation. This technology includes antimicrobial agents sourced from spice by-products into packaging materials, forming barriers that prevent microbial growth on food surfaces [211]. Phenolic acids, flavonoids, essential oils, and alkaloids extracted from spice processing waste are typically released through diffusion, dissolution, or enzymatic degradation when incorporated into food matrices or packaging materials [212]. Once released, they disrupt microbial cell membranes by altering membrane permeability and fluidity, inhibiting key enzymatic processes essential for cellular metabolism, and interfering with DNA replication and protein synthesis [213] (Figure 4A,B).

This approach provides advantages over traditional preservation methods by ensuring the uniform distribution of antimicrobial agents throughout the packaging matrix. The shift toward natural antimicrobials derived from spice by-products addresses growing concerns about microbial resistance to synthetic preservatives while aligning with consumer preferences for natural food packaging systems [214].

Plant-based antimicrobials from spice by-products consist primarily of polyphenols and essential oils that effectively target food pathogens while maintaining packaging biodegradability [215]. Polyphenols from industrial by-products, like pepper pomace, garlic, and onion peels, have shown efficacy against common spoilage organisms [216,217] (Figure 4C,D). Recent studies have demonstrated the efficacy of these systems in commercial applications. Red onion and shallot tunic extracts incorporated into edible films significantly reduced counts of *E. coli*, coliforms, and *Staphylococcus aureus* on fresh beef tenderloin for up to seven days compared to uncoated controls [218]. These natural antimicrobial packaging systems enhance food safety and improve the economic value of agricultural operations by utilizing previously discarded materials [219].

### 4.3. Shelf-Life Extension Packaging Systems

Effective biodegradable packaging systems must address multiple preservation challenges, including moisture control, microbial inhibition, and oxidation prevention. Recent studies have demonstrated how spice by-products can be effectively integrated into complete packaging systems that significantly extend the food shelf life. Clove essential oil emulsions combined with arrowroot starch and beeswax film have enhanced tomato preservation, extending the shelf life to 49 days while maintaining physicochemical characteristics [220]. Similarly, carvacrol from thyme and oregano oils incorporated into gelatin/carboxymethyl chitosan films extended the shelf life of Chinese mitten crab by three days at 4 °C through combined antimicrobial and antioxidant mechanisms [221]. These systems establish how spice by-products can preserve food and reduce waste via eco-friendly packaging.

### 4.4. Barrier Properties

Biodegradable packaging materials from spice by-products offer dual benefits: practical preservation barriers and environmental sustainability. These materials regulate moisture and oxygen transmission, which are critical factors that prevent food spoilage [222]. Hydrocolloids extracted from spice by-products, including mucilage and other polysaccharides, create practical moisture barriers when incorporated into packaging films. A study conducted by Mohite et al. [223] demonstrated exceptional water impermeability by a novel fenugreek seed mucilage-taro starch film. The results indicated that water vapor permeability ranged from 1.53 mg/Pa·s·m^2^ × 10^−11^ to 2.79 mg/Pa·s·m^2^ × 10^−11^, with the permeability observed in the order of F0 > T1 > T2 films. The research identified various combinations of hydrogen ions, alongside the hydrophilic and hydrophobic characteristics linked covalently among the film particles. This tightly structured arrangement renders the film suitable for the development of edible films.

### 4.5. Controlled Release Systems

Advanced packaging systems incorporating spice by-products utilize controlled release mechanisms to optimize preservation effects throughout the product shelf life. These systems embed bioactive compounds within polymer matrices that gradually release active components in response to environmental triggers or over predetermined timeframes [224].

Essential oils from spice by-products demonstrate characteristic release patterns when incorporated into biodegradable films. Investigations on the essential oil of *T. vulgaris* in pectin films reveal two distinct phases of release: an initial burst followed by a prolonged release period [225]. The choice of surfactant significantly affects the release kinetics, with Pluronic F127 showing superior performance over Tween 80 by decreasing the volatility of the essential oil while enhancing its antioxidant efficacy. Multiple elements influence the release dynamics, including the makeup of the polymer matrix, the conditions during processing, such as sonication and temperature, and environmental aspects, like storage conditions.

**Figure 5:**
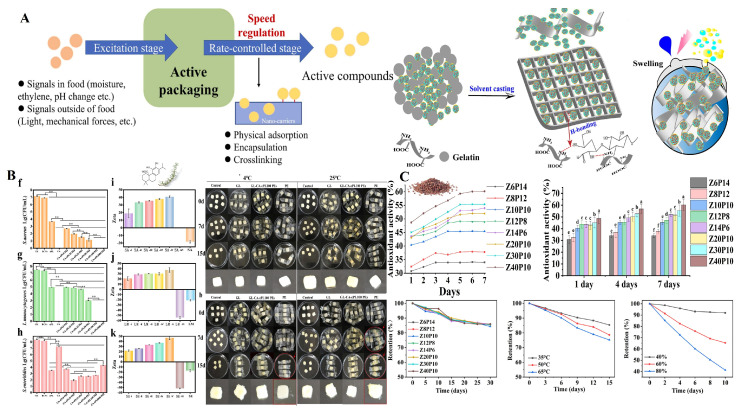
Role and benefits of spice by-products in controlled release systems for active packaging (**A**) Schematic representation of controlled release mechanisms of active compounds in active packaging. Reproduced with permission [228] (**B**) Antimicrobial efficacy evaluation through zone of inhibition assays (f–k) and release kinetics analysis at varying temperatures, showing sustained compound release profiles of three-phase Pickering nanoemulsion system containing rosemary leaves-carnosic acid. ** *p* < 0.01. Reproduced with permission [226] (**C**) Slow release film containing *Zanthoxylum* residue extract showing release kinetics over 7 days, cumulative release profiles at different time intervals, and temperature-dependent release behavior, demonstrating controlled delivery of bioactive compounds for enhanced food preservation applications. a–f means in the same column with different letters differ significantly (*p* < 0.05). Reproduced with permission [227].

Pickering emulsions, moreover, represent a promising approach for developing controlled nutrient-release systems. Research by Xia et al. [226] and Qin et al. [227] highlighted the potential of these emulsions as humidity-sensitive mechanisms for the delivery of nutrients. These studies emphasize the efficacy of emulsions in encapsulating bioactive substances derived from rosemary and Szechuan pepper waste, facilitating controlled and prolonged release of antimicrobial and antioxidant compounds as needed. Integrating phytochemicals derived from plant biomass addresses the challenge of achieving stability and controlled release of essential plant oils in Pickering emulsions (Figure 5).

Zhang et al. [229] illustrated the potential for the controlled release of spice bioactives by creating a ginger essential oil Pickering emulsion within a sodium alginate film. The antioxidants and antimicrobial substances in this film were released gradually, providing ongoing protection against spoilage-related microbes and oxidation while preserving the high postharvest quality of mango fruits. This highlights how nanoscale structural alterations can greatly influence preservation effectiveness at a macroscopic level. Additionally, in a recent study by Rahman et al. [230], the preservation characteristics of *Piper chaba* stem extracts, a well-known Asian spice, were investigated applying them to beef patties, where it showed slower degradation of fat and enhanced oxidative stability over a period of time of 33 days, compared to the control group. This practical application demonstrates the real-world effectiveness of controlled release systems in food preservation. Table 4 outlines the significant implications of incorporating spice by-products into food packaging systems.

## 5. Challenges and Future Perspectives

The incorporation of agricultural by-products is steadily growing into a pivotal point of focus across multiple industries, particularly within the food packaging sector. As a result, the utilization of spice by-products for active food packaging is gaining traction.

Spice residues are less hazardous than non-degradable polymers, but they face challenges in their use. A major concern is the long-term stability of bioactive compounds, such as anthocyanins and phenolics, which can diminish when exposed to heat, oxygen, and light. Moreover, interactions with food and variable release kinetics impact the shelf life of the product [243].

Incorporating spice by-products into packaging materials poses challenges related to mechanical properties that are essential for commercial viability. A significant challenge associated with incorporating spice by-products is the potential decline in tensile strength, occurring due to inconsistent stress distribution and the introduction of weak points within the material. Spice by-product-based films typically exhibit a tensile strength below the 30 MPa threshold required by packaging standards, with reduced flexibility and moisture resistance compared to polyethylene or polypropylene. Such changes can adversely affect the crystallinity and mobility of polymer chains, leading to a decline in overall material performance [244].

Due to complex processing, spice by-product packaging costs 20–30% more than conventional options. Scaling from lab to industrial production is challenging due to raw material variability and the need for stability in manufacturing. There is significant potential to improve the efficiency of agro-biorefineries through the strategic application of microorganisms and enzymes [245].

Encapsulation technology offers solutions through protective mechanisms, such as physical barriers, controlled release systems, and enhanced dispersibility in packaging. Key physical methods include spray drying, freeze drying, fluid bed coating, and extrusion. Additionally, chemical strategies, like complex coacervation, micro- and nanoencapsulation, liposomal systems, and emerging technologies, such as intelligent polymeric systems and biodegradable materials, are driving advancements in spice processing and preservation [246].

While foundational regulatory frameworks exist (EC 1935/2004, EC 450/2009), their application to bio-based materials incorporating bioactive compounds presents unique challenges. The multi-component nature of spice by-product extracts requires case-by-case evaluation, potentially extending approval timelines and increasing development costs. Therefore, collaborative establishment of standardized testing protocols, safety assessment methods, and certification systems tailored for bioactive compound-containing packaging materials is required [247].

## 6. Conclusions

Spice by-products represent a valuable resource for developing sustainable active food packaging systems. These materials exhibit unique multifunctional properties through their rich phytochemical profiles, including phenolics, essential oils, polysaccharides, and dietary fibers, which provide controlled release antimicrobial and antioxidant activities. The bioactive compounds retained in spice processing waste show superior performance compared to typical plant residues, with ginger and onion residues being the most viable, delivering enhanced antioxidant capacity while cinnamon and garlic waste show great potential for improved mechanical strength and barrier properties.

The incorporation of spice by-products into biodegradable films, coatings, and nanoemulsions has proven feasible through green extraction technologies. However, commercialization faces significant challenges, including bioactive compound instability during extended storage, mechanical durability limitations during scale-up, natural compositional variability affecting standardization, and cost competitiveness with conventional packaging materials.

Several technological solutions show promise for overcoming these barriers. However, encapsulation techniques, nano-reinforcement strategies, and intelligent polymeric systems are the most promising for improving bioactive stability and functional performance. The regulatory landscape, governed by frameworks, such as EC 1935/2004 and EC 450/2009, also requires the development of streamlined approval pathways for bio-based packaging materials containing plant-based compounds.

Future research priorities should focus on establishing standardized characterization protocols for spice by-products across different sources and processing methods, validating industrial-scale production feasibility, developing cost-effective extraction and processing technologies, and creating regulatory guidelines specific to bioactive packaging materials. Commercial implementation may be most viable initially in high-value food sectors where clean-label and natural preservation are prioritized.

In conclusion, the strategic use of spice by-products in active packaging represents a significant opportunity to advance circular bio-economy principles, reduce dependence on petroleum-based materials, and contribute to sustainable food system development while addressing the growing challenge of agro-industrial waste management.

## Figures and Tables

**Table 1:** Various by-products streams mostly found in spice processing.

Spice	Botanical Name	Waste Parts/By-Products	References
Turmeric 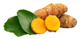	*Curcuma longa* L.	Leaves, spent residue, press residue	[19,20]
Pepper 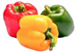	*Capsicum* spp.	Seeds, defective fruits, spent residue	[21]
Saffron 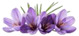	*Crocus sativus* L.	Leaves, petals, stamens, defective stigmas	[22]
Coriander 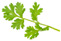	*Coriandrum sativum* L.	Spent residue, roots, bark, seeds	[23]
Ginger 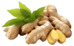	*Zingiber officinale*	Skin/peel, stems, stalk, leaves, herbal dust, spent residue	[24]
Garlic 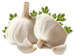	*Allium sativum*	Husks/peels, straws, stem, leaves	[25]
Onion 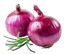	*Allium cepa*	Skin, roots, top and bottom bulbs, smashed/deformed onions	[26]
Clove 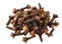	*Syzygium aromaticum*	Leaves, spent residue, stem	[27]
Szechuan Pepper 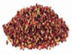	*Zanthoxylum* spp.	Leaves, seeds, branches	[28,29]
Cumin 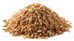	*Cuminum cyminum*	Oilseed cake	[30]

**Table 2:** Nutritional and chemical composition of by-product streams of commonly used spices.

Spice	Botanical Name	By-Products	Chemical Composition (%)										References
			Ash	Protein	Fat	Starch	Pectin	Cellulose	Sugars	Hemicellulose and Lignin	Uronic Acids	Fiber	
Ginger	*Zingiber officinale*	Stem and Leaves	7.04	6.02	-	-	-	48.48	52.14	31.50	-	-	[34]
		Spent Residue	9.37	11.74	1.67	50.79	2.09	3.84	4.52	-	-	-	[34]
		Pomace	-	8.33	-	16.62	-	-	76.42	-	4.42	-	[35]
Turmeric	*Curcuma longa* L.	Dye Extraction Residue	5.80	4.20	-	64.00	-	8.30	-	13.10	-	21.00	[36]
		Juice Extraction Press Residue	-	7.40	5.98	-	-	-	42.46	-	-	5.75	[19]
		Spent turmeric powder	26.04	12.67	-	-	-	-	39.66	-	-	-	[37]
		Leaves	9.4	6.0	0.5	-	-	-	-	44.74	-	34.5	[38]
Pepper	*Capsicum* spp.	Seeds	3.05	28.33	18.39	-	-	-	-	-	-	-	[39]
		Pomace	5.67	15.52	10.80	1.78	6.40	18.25	9.58	23.55	-	48.27	[40]
Coriander	*Coriandrum sativum* L.	Flowers	3.52	11.94	6.19	-	-	-	-	-	-	8.41	[41]
Saffron	*Crocus sativus* L.	Petals	12.4	21.7	14.1	-	-	-	-	-	-	28.9	[42]
Garlic	*Allium sativum*	Biomass	2.92	13.09	-	-	-	25.37	83.99	47.73	-	-	[43]
		Straw	10.30	3.32	4.59	-	-	-	-	6.68	-	25.80	[44]
		Peel	7.37	2.61	0.22	8.5	26.00	18.62	7.87	26.93	-	62.10	[45]

**Table 3:** Abundant phenolic compounds in solid waste residues and by-product streams of commonly utilized spices.

Spice Waste/By-Product	Extraction Conditions	Main Phenolic Compounds	Reference
Saffron flowers 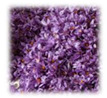	Solvent extraction: MeOH:H_2_O (7:3 *v*/*v*, 1:10 *w*/*v*), 37 °C, 24 h	3-Hydroxytyrosol (C_8_H_10_O_3_) Epicatechin (C_15_H_14_O_6_) Vanillic Acid (C_8_H_8_O_4_) Rosmarinic Acid (C_18_H_16_O_8_) Gallic Acid (C_7_H_6_O_5_) Quercetin (C_15_H_10_O_7_) 4-Hydroxybenzoic Acid (C_7_H_6_O_3_) Chlorogenic Acid (C_16_H_18_O_9_) Myricetin (C_15_H_10_O_8_) Salicylic Acid (C_7_H_6_O_3_)	[61]
Saffron defective stigmas 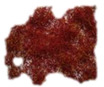	(a) UAE: EtOH: H_2_O (100% H_2_O, 50:50, 96:4% *v*/*v*), (20, 60, 100) amplitude, 3 min; (b) SWE: EtOH: H_2_O (50:50 and 96:4% *v*/*v*), 125–250 °C, 20 min; (c) DES extraction: Choline chloride:16 varying HBDs, (80:20 *v*/*v*), 2 min	Coumaric Acid (C_9_H_8_O_3_) Rosmarinic Acid (C_18_H_16_O_8_) Vanillic Acid (C_8_H_8_O_4_) Caffeic Acid (C_9_H_8_O_4_) Gallic Acid (C_7_H_6_O_5_) Hydroxycinnamic Acids (C_9_H_8_O_3_) Syringic Acid (C_9_H_10_O_5_) Hydroxybenzoic Acid (C_7_H_6_O_3_) Rutin (C_27_H_30_O_16_) Naringin (C_27_H_32_O_14_) Catechin (C_15_H_14_O_6_) Isovanillin (C_8_H_8_O_3_) Epicatechin (C_15_H_14_O_6_) Kaempferol (C_15_H_10_O_6_)	[62]
Saffron processing residue 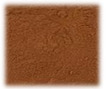	DES extraction: L-lactic acid: Glycine (55–85% *w*/*v*), 50 °C, 150 min	Kaempferol-3-*O*-Sophoroside (C_27_H_30_O_16_) Isorhamnetin Glucosides (C_22_H_22_O_12_) Quercetin (C_15_H_10_O_7_)	[63]
Sage residue from steam distillation 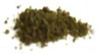	UAE: MeOH: H_2_O (7:3 *v*/*v*, 1:10 *w*/*v*), 30 °C, 15 min	Vicenin-2 (C_27_H_30_O_15_) Epigallocatechin (C_15_H_14_O_7_) Luteolin-7-O-Rutinoside (C_27_H_30_O_16_) Luteolin-7-O-Glucoside (C_21_H_20_O_11_) Verbascoside (C_29_H_36_O_15_) Isorhamnetin-3-Rutinoside (C_28_H_32_O_16_) Apigenin-7-O-Glucoside (C_21_H_20_O_10_)	[64]
Sage dust 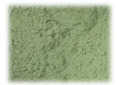	Soxhlet extraction: Methylene chloride/Hexane (120 mL each, 12:1 *w*/*v*), 40 °C, 6 h	Caffeic Acid (C_9_H_8_O_4_) Hydroxybenzoic Acid (C_7_H_6_O_3_) Ferulic Acid (C_10_H_10_O_4_) Rosmarinic Acid (C_18_H_16_O_8_)	[33]
Sage dry leaves 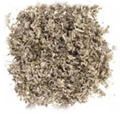	UAE: H_2_O/EtOH/MeOH (1:10 *w*/*v*), 20–40 °C, 15–300 min	Ferulic Acid (C_10_H_10_O_4_) Ellagic Acid (C_14_H_6_O_8_) Caffeic Acid (C_9_H_8_O_4_) *p*-Hydroxybenzoic Acid (C_7_H_6_O_3_) Vanillic Acid (C_8_H_8_O_4_) Syringic Acid (C_9_H_10_O_5_) *p*-Coumaric Acid (C_9_H_8_O_3_) Chlorogenic Acid (C_16_H_18_O_9_) 3-Hydroxybenzoic Acid (C_7_H_6_O_3_) 3,4-Hydroxybenzoic Acid (C_24_H_20_O_7_) Luteolin (C_15_H_10_O_6_) Myricetin (C_15_H_10_O_8_) Chrysin (C_15_H_10_O_4_) Kaempferol (C_15_H_10_O_6_) Galangin (C_15_H_10_O_5_)	[65]
Turmeric leaves 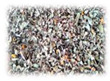	Solvent extraction: Distilled water (1:25 *w*/*v*), 85 °C, 150 min	Rutin (C_27_H_30_O_16_) Quercitrin (C_21_H_20_O_11_) Myricetin-3-O-rhamnoside (C_21_H_20_O_12_) Quercetin (C_15_H_10_O_7_) Diosmetin (C_16_H_12_O_6_) Miquelianin (C_21_H_18_O_13_)	[60]
Garlic husks 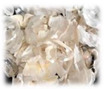	(a) Carbon dioxide expanded extraction: EtOH, 50–200 °C, 0.5–2 mL/min, 2 h; (b) Pressurized extraction: 150 °C, 10 MPa, 3 mL/min ethanol flowrate, 2 h; (c) Soxhlet extraction: MeOH and EtOH (1:100 *w*/*v)*, 6 h	Gallic Acid (C_7_H_6_O_5_) *Trans*-ferulic Acid (C_10_H_10_O_4_) 4-Hydroxybenzoic Acid (C_7_H_6_O_3_) Caffeic Acid (C_9_H_8_O_4_) Hydroxybenzoic Acid (C_7_H_6_O_3_)	[56]
Coriander seeds 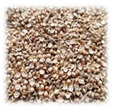	(a) NaDES-assisted-maceration: Aqueous MeOH (1:20 *w*/*v*), 25 °C, 20/40 min, 2, 4, and 6 h; (b) UAE-NaDES extraction: (1:20 *w*/*v*), 70 W, 25 °C, 30 min	Catechin (C_15_H_14_O_6_) 3,4-Dimethoxycinnamic Acid (C_11_H_12_O_4_) Coumaric Acid (C_9_H_8_O_3_) Daidzein (C_15_H_10_O_4_) Ferulic Acid (C_10_H_10_O_4_) Sinapic Acid (C_11_H_12_O_5_) *Trans*-ferulic Acid (C_10_H_10_O_4_) Chlorogenic Acid (C_16_H_18_O_9_) Neochlorogenic Acid (C_16_H_18_O_9_) Luteolin (C_15_H_10_O_6_) Kaempferol (C_15_H_10_O_6_) Quercetin (C_15_H_10_O_7_)	[66]
Coriander seed cake 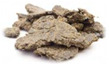	Solvent extraction: 100% MeOH (1:4 *w*/*v*), room temperature, 30 min	Eugenol (C_10_H_12_O_2_)	[67]
Szechuan pepper leaves 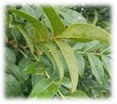	MAE: 65% EtOH (1:30 *w*/*v*), 500 W, 70 °C, 4 min	Quercetin (C_15_H_10_O_7_) Quercetin-3-O-Glucoside (C_21_H_20_O_12_) Hyperoside (C_21_H_20_O_12_) Rutin (C_27_H_30_O_16_) Isoquercetin (C_21_H_20_O_12_) Kaempferol-3-O-Galactoside (C_21_H_20_O_11_) Diosmetin (C_16_H_12_O_6_) Apigenin (C_15_H_10_O_5_) Catechin (C_15_H_14_O_6_) Epicatechin (C_15_H_14_O_6_) Chlorogenic Acid (C_16_H_18_O_9_) Protocatechuic Acid (C_7_H_6_O_4_) Caffeic Acid (C_9_H_8_O_4_)	[68,69]
Szechuan pepper twigs 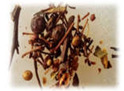	Solvent extraction: MeOH/Dichloromethane/Ethylacetate/n-Butyl alcohol	*Trans*-Ferulic Acid (C_10_H_10_O_4_) Methyl Ferulate (C_11_H_12_O_4_) Vanillic Acid (C_8_H_8_O_4_) Methyl *p*-Hydroxycinnamate (C_10_H_10_O_3_) Eugenol (C_10_H_12_O_2_) Methyl Syringate (C_10_H_12_O_5_)	[70]
Ginger peel 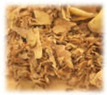	UAE: EtOH (1:11.25 *w*/*v*), room temperature, 15 min	Rutin (C_27_H_30_O_16_) Zingerone (C_11_H_14_O_3_) Quercetin (C_15_H_10_O_7_) Kaempferol (C_15_H_10_O_6_) Naringenin (C_15_H_12_O_5_) 6-Gingerone (C_17_H_26_O_3_)	[71]
Ginger leaves 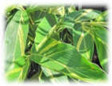	Hot-water extraction: H_2_O (1:10 *w*/*v*), 90 ± 2 °C, 60 min	Kaempferol (C_15_H_10_O_6_) Quercetin (C_15_H_10_O_7_) 3-*O*-Robinobioside-7-*O*-Rhamnoside (C_66_H_128_O_19_Si_11_) 3-*O*-Galactoside-7-*O*-Rhamnoside (C_27_H_30_O_15_)	[59]
Onion peel 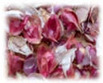	Solvent extraction: 80% MeOH (1:10 *w*/*v)*, 33 ± 3 °C, 24 h	Vanillic Acid (C_8_H_8_O_4_) Quercetin (C_15_H_10_O_7_) Cyanidin (C_15_H_11_O_6_^+^)	[72]
Clove aerial parts 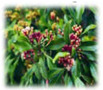	Maceration: EtOH (1:1; *v*/*v*, 500 mL), room temperature, 72 h	Eugenol (C_10_H_12_O_2_) Eugenyl Acetate (C_12_H_14_O_3_) 4-(2-Propenyl)-Phenol (C_9_H_10_O) Gallic Acid (C_7_H_6_O_5_)	[73]
Thyme herbal dust 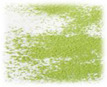	(a) SFE: CO_2_, 15 bar, 25 °C, 3 h; (b) Soxhlet extraction: Hexane/Methylene chloride (120 mL each, 1:12 *w*/*v*), 6 h	Protocatechuic Acid (C_7_H_6_O_4_) Vanillic Acid (C_8_H_8_O_4_) Coumaroyl Hexoside (C_15_H_18_O_8_) Luteolin (C_15_H_10_O_6_) Kaempferol-3-Rutinoside (C_27_H_30_O_15_)	[74]

**Table 4:** Application of bioactive spice by-products in food packaging systems and key findings.

Spice By-Product	By-Product Bioactive Compounds	Film Preparation Method(s) and Conditions	Biocomposite Composition	Key Findings in Food Packaging Systems	Food Matrix Application	References
Turmeric extraction dye residue	Curcuminoids Starch Fiber	Solvent casting, Oven drying; 35 °C, 7 h	Turmeric starch Glycerol	• Antioxidant activity (46% DPPH scavenging vs. 74% in raw residue) delayed lipid oxidation and extends food shelf life by 20–30% • Optimized films showed low WVP of 0.296 g.mm.h^−1^.m^−2^.kPa^−1^; 27% higher than control films • Increased TS of 8.9 MPa comparable to synthetic LDPE films • YM of 540–644 MPa; stiffness similar to polystyrene but brittle for flexible applications • Curcuminoids retained at 5.81 mg/L bisdemethoxycurcumin reduced *E. coli* and *S. aureus* by 1–2 log CFU/mL in 24 h	N/A	[231]
Ginger starch residue	Starch	Solvent casting, Oven drying; 50 °C, 72 h	Keratin feather Chia seed oil Glycerol	• Shelf life extended up to 21 days at 25 °C, whereas uncoated tomatoes deteriorate by day 12–15 • 63.2% film transparency enhanced photo-oxidation resistance • 12.6% lower WVP of 0.0277 g.mm.h^−1^.m^−2^.kPa^−1^ was noticed indicating good moisture barrier • Improved TS of 8.15 MPa and 47.4% increase in EAB indicated improved flexibility • Improved thermal stability of 310.98 °C vs. 298.66 °C (control), indicated high heat resistance • Preservation efficacy was enhanced by 30% reduction in PPO activity	Coating on tomato fruits	[232]
Ginger waste	Nanocellulose	Solvent casting, Oven drying; 60 °C, 24 h	Sodium Alginate (SA) Chitosan (CS)	• TS in SA films increased by 94% with 5% GNC, EAB improved by 56–89% at 1–5% GNC and YM rose from 81.56 MPa to 92.68 MPa at 5% GNC • TS in CS films were enhanced by 64% with 5% GNC and EAB decreased by 21–30%, showing increased rigidity • WCA increased but remained <90°, maintaining hydrophilic character • GNC improved thermal stability of CS films • Low GNC concentrations yielded uniform and defect-free films while high concentrations caused agglomeration, reducing performance	N/A	[233]
Garlic waste	Quantum Carbon Dots (CDs)	Solvent casting, Oven drying; 60 °C, 3 h	Pectin Glycerol	• TS increased by 92% from 3.62 MPa to 6.96 MPa with CDs incorporation • EAB improved by 148% proving enhanced flexibility • DPPH scavenging capacity doubled at 300 μg/mL film concentration • CDs/pectin film reduced salmonella survival to 5% at 300 μg/mL film concentration • Moisture barrier performance was maintained at a minimal change of 1.06 × 10 g.m^−1^.h^−1^.Pa^−1^ • CDs showed fluorescence quenching under acidic conditions, providing good spoilage detection • Reduced water loss and no mold growth on strawberries after 5 days vs. severe decay in uncoated controls was observed • Significant reduction in surface CFUs compared to controls was observed	Coating on strawberries	[234]
Garlic peels	Polyphenols	Solvent casting, Oven drying; 40 °C, 24 h	Alginate Polyvinyl alcohol Nelumbo nucifera (lotus) flower extract	• WVP improved from 11.93 × 10^−11^ to lower values, enhancing moisture barrier performance • OTR reduced with higher extract concentration; attributed to hydrogen bonding and denser film structure • Weight loss reduction from 88% to 72%, showed improved heat resistance • Extended shrimp shelf life at room temperature • >70% degradation within 120 days, indicating good sustainability	Shrimp freshness	[235]
Garlic waste peel	Polyphenols	Solvent casting, Oven drying; 60 °C, 5 h	Chitosan	• Decreased WVP from 657.3 ± 0.57 g/m^2^/day to 640.4 ± 0.58 g/m^2^/day, proving improved barrier properties • TS increased from 12.5 MPa to 30 MPa with polyphenol addition • YM rose significantly from 0.341 GPa to 0.77 GPa, reflecting durability and better stiffness • Antioxidant capacity was enhanced greatly from 14.80% to 40.63% for better food oxidation prevention • Significant microbial inhibition zone of 0.65 cm and 0.5 cm for *K. pneumoniae* and *S. aureus* • Slight increase of thermal stability with polyphenol addition • Excellent UV protection factor • Observed non-toxicity and biocompatibility with L929 mouse connective tissue cells	Red Apples	[236]
Garlic skin	Polyphenols CNCs	Solvent casting, Oven drying; 37 °C, 72 h	Chitosan	• A 74% improvement observed in TS of CNC incorporated films • Increase in YM of CNC incorporated film from 909.45 MPa to ~1500 MPa • Improved UV transmittance as opacity of films increase with increasing CNC and polyphenol addition • Thermal stability increased from 277.3 °C to 288.9 °C • CNC and polyphenol films degraded by 55.69% after 70 days of soil burial • >80% antibacterial activity recorded against *S. aureus* and *S. alboviridis*		[168]
Garlic skin	Polyphenols	Solvent casting, Oven drying; 60 °C, 15 h	Potato Starch Gellan Gum Glycerol	• Enhanced mechanical properties with YM ranging from 122 MPa to 454 MPa and total stress peaking at 1.70 MPa • Microstructure of films improved at high concentration of polyphenol addition • Improved water solubility and sensitivity with finer garlic skin particles • Films degraded within 60 days showing sustainability • Phenolic content increased from 0.019 mg GAE/g to 0.051 mg GAE/g with particle refinement	N/A	[237]
Szechuan pepper seeds	Nisin	Water-in-oil-in-water (W/O/W) microencapsulation technique, 25 °C, 15 min	Gum Arabic	• Microcapsules demonstrated strong antimicrobial effects (89.75% against *S. aureus* and 81.33% against *E. coli*) • Shelf life of coated peppers was extended by 10 days • High TPC of 543.56 mg GAE/g was recorded in 3:1 ratio of protein to gum arabic • Zeta potential of up to −27.03 ± 0.67 mV indicated stable microcapsules with reduced aggregation risk	Coating on szechuan peppers	[238]
Black pepper grains	Piperine	Compression molding; Cooling, 25 °C	PLA	• Crystallinity improved from 2.6% untreated PLA to 37.5% treated film, improving thermal stability • YM decreased by up to 65% and EAB increased to 12.3%, showing better flexibility • SEM and AFM showed increased surface roughness of up to 131.6 nm, aiding controlled release • High crystallinity improved film transparency	N/A	[239]
Cumin defatted seed cake	Proteins	Solvent casting, Oven drying; 37 °C, 48 h	Glycerol Transglutaminase (TGase)	• Low YM of 18.7 MPa confirmed flexibility • Low VP of 0.002 g mm m^−2^ day^−1^ kPa^−1^ was maintained, demonstrating good moisture resistance • Films were effective against *S. aureus* inhibition • SEM showed smooth surface and denser cross-sections in all films, improving the mechanical and barrier properties	N/A	[240]
Coriander straw	Fiber	Twin-screw extrusion compounding, Injection molding; 100 mm/s, 2000 bar, 20 °C, 15 s	Polypropylene (PP) Biobased low-density polyethylene (LDPE)	• Minimal moisture absorption (<5% density charge) and oxidative stability due to lignin properties • Higher flexural strength was retained post UV exposure • <10% loss in TS after 5 reprocessing cycles	N/A	[241]
Szechuan pepper seed	Soluble Dietary fiber (SDF)	Complex coacervation and Spray drying, pH 4.1, SPI-to-SDF mass ratio 6:1	Soybean protein isolate (SPI)	• Delayed bacterial growth >12 h • Enhanced thermal/storage stability (225 °C resistance and 80% retention at 45 °C) • Preserved bioactivity after encapsulation was recorded with DPPH scavenging of 50% at 7.67 mg/mL • Improved release kinetics as the fastest release of bioactives was noticed in 50% ethanol and slowest in water • Encapsulated essential oil retained >80% of its initial content still after 15 days at 45 °C)	Szechuan pepper essential oil	[242]

## Data Availability

No new data were created or analyzed in this study. Data sharing is not applicable to this article.

## Abbreviations

EFCs	edible films and coatings
MIC	minimum inhibitory concentration
PCs	phenolic compounds
GPPs	ginger peel polysaccharides
BPs	bioactive peptides
POD	peroxidases
PPO	polyphenol oxidases
CNCs	cellulose nanocrystals
SWE	subcritical water extraction
SFE	supercritical fluid extraction
VOCs	volatile organic compounds
UAE	ultrasound-assisted extraction
MAE	Microwave-assisted extraction
PLE	pressurized liquid extraction
EAE	Enzyme-assisted extraction
HC	hydrodynamic cavitation
NaOH	sodium hydroxide
SPI	soy protein isolate
SDF	soluble dietary fiber
LDPE	low density polyethylene
PP	polypropylene
W/O/W	water-in-oil-in-water
TPC	total polyphenol content
DPPH	2,2-diphenyl-1-picrylhydrazyl
TVC	total viable count
ORAC	oxygen radical absorbance capacity
RSTE	red onions shallot tunic extracts
GL/CC	gelatin/carboxymethylcellulose
CA	carvacrol
TVB-N	total volatile base nitrogen
PLA	poly lactic acid
ABTS	2,2′-azino-bis(3-ethylbenzothiazoline-6-sulfonic acid)
PFE	pulsed electric field extraction
HPP	high pressure processing
NaDESs	Natural deep eutectic solvent
DES	deep eutectic solvent
EtOH	ethanol
MeOH	methanol
L/S	liquid to solid ratio
WVP	water vapor permeability
YM	Young’s modulus
TS	tensile strength
CFU	colony forming unit
GNC	ginger waste nanocellulose
WCA	water contact angle
OTR	oxygen transmission rate
